# Coffee and cashew nut dataset: A dataset for detection, classification, and yield estimation for machine learning applications

**DOI:** 10.1016/j.dib.2023.109952

**Published:** 2023-12-14

**Authors:** Rahman Sanya, Ann Lisa Nabiryo, Jeremy Francis Tusubira, Sudi Murindanyi, Andrew Katumba, Joyce Nakatumba-Nabende

**Affiliations:** a Department of Adult and Community Education, Makerere University, Kampala, Uganda; b Department of Computer Science, Makerere University, Kampala, Uganda; c Department of Electrical and Computer Engineering, Makerere University, Kampala Uganda

**Keywords:** Coffee cherry, Cashew apple, Object detection, Image classification, Yield estimation

## Abstract

Conventional methods of crop yield estimation are costly, inefficient, and prone to error resulting in poor yield estimates. This affects the ability of farmers to appropriately plan and manage their crop production pipelines and market processes. There is therefore a need to develop automated methods of crop yield estimation. However, the development of accurate machine-learning methods for crop yield estimation depends on the availability of appropriate datasets. There is a lack of such datasets, especially in sub-Saharan Africa. We present curated image datasets of coffee and cashew nuts acquired in Uganda during two crop harvest seasons. The datasets were collected over nine months, from September 2022 to May 2023. The data was collected using a high-resolution camera mounted on an Unmanned Aerial Vehicle . The datasets contain 3000 coffee and 3086 cashew nut images, constituting 6086 images. Annotated objects of interest in the coffee dataset consist of five classes namely: unripe, ripening, ripe, spoilt, and coffee_tree. Annotated objects of interest in the cashew nut dataset consist of six classes namely: tree, flower, premature, unripe, ripe, and spoilt. The datasets may be used for various machine-learning tasks including flowering intensity estimation, fruit maturity stage analysis, disease diagnosis, crop variety identification, and yield estimation.

Specifications Table

**Table:** 

Subject	Computer Vision, Applied Machine Learning, Agriculture
Specific subject area	Remote sensing with UAV, object detection, image classification, precision agriculture, yield estimation
Type of data	Image
Data format	Raw images
Description of data collection	The datasets were collected during harvest seasons for coffee and cashew over nine months in 2022 and 2023. The imaging equipment used consisted of 20/48-megapixel cameras mounted on a DJI Mini 3 Pro UAV or drone with a 1/1.3 in CMOS sensor, lens with aperture of f/1.7 and focus range of 1 m to ∞, shutter speed of 2-1/8000s and ISO range of 100-6400 (Auto and Manual)
Data source location	Institution: Makerere University City: Kampala Country: Uganda
Data accessibility	Repository name: Mendely Data Data identification number: http://doi.org/10.17632/r46c6bpfpf.1 Direct URL to data: https://data.mendeley.com/datasets/r46c6bpfpf/1

## 1 Value of the Data

• Flowering intensity estimation. Flowering represents an important stage in coffee and cashew farming since it affects crop yield. It has a significant impact on yield in that flowering intensity is positively correlated with the amount of crop yield. Therefore, flowering intensity could be an important predictor of crop yield [1]. Our dataset contains a flowering class, which can be used to train machine learning models to estimate the flowering intensity of cashew crops.
• Fruit detection. In crop yield estimation using computer vision techniques, accurately detecting objects of interest, e.g., fruit pods is critical. Our dataset can be used to train machine learning models for the automated detection of coffee cherries and cashew apples. The dataset may also facilitate the development of new algorithms for small object detection, currently an open research problem in computer vision, since coffee cherries and cashew apples are relatively small.
• Fruit maturity stage analysis. Our datasets contain coffee cherries and cashew apples at various stages of growth or maturity. It is these maturity stages that constitute object classes in the datasets. The dataset can be used to build machine learning models for automated analysis of fruit maturity stages for various purposes, including harvest scheduling.
• Coffee crop variety identification. Our coffee dataset features the two main varieties grown in Uganda, namely Robusta (*Coffea canephora*) and Arabica (*Coffea arabica*) [2]. Within the Robusta variety, there are at least ten Coffee Wilt Disease resistant (CWD-r) clones, also known as KR lines. These clones are also resistant to leaf rust, tolerant to Red Blister Disease (RBD), have larger coffee bean sizes, are higher yielding, and have better cup quality. This means that our dataset can potentially be used for building models for the automated identification of Robusta coffee varieties.
• Crop yield estimation. Our coffee and cashew datasets may also be used for yield estimation using machine learning methods, similar to work in [3], [4], [5]. Various machine-learning approaches may be used for yield estimation with this dataset, including object detection, image-based regression, and vegetation index-based methods.
• Fruit disease diagnosis. Coffee cherries and cashew apples belong to three and five classes, namely unripe, ripe, spoiled and flower, immature, unripe, ripe, and spoiled, respectively. Images belonging to the spoiled class in each dataset may be used for coffee and cashew fruit disease diagnosis.

## 2 Data Description

The datasets presented in this work consist of high-resolution images of coffee and cashew plants acquired using Unmanned Aerial Equipment (UAV) equipment from small and large-scale farms across Uganda. Images range approximately between 10 MB and 12 MB in size, approx. 4000 by 3200 pixels in dimension and 72 pixels/in in Dots per inch (DPI). Each image is annotated with multiple bounding boxes, each enclosing an object of interest. Each image is accompanied by metadata, including the date (timestamp) and the geographic location (latitude and longitude) where it was captured.

**Fig. 1:**
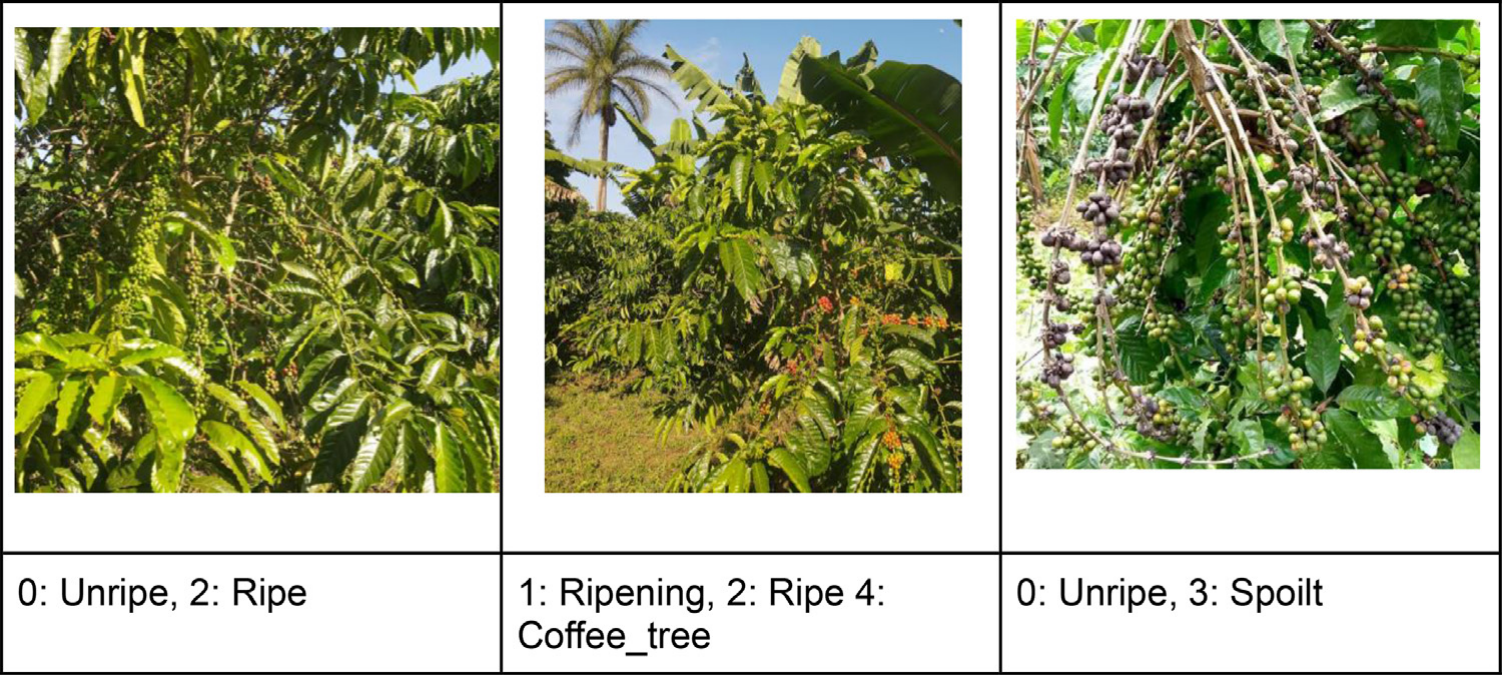
Sample coffee images showing the different coffee class labels.

**Fig. 2:**
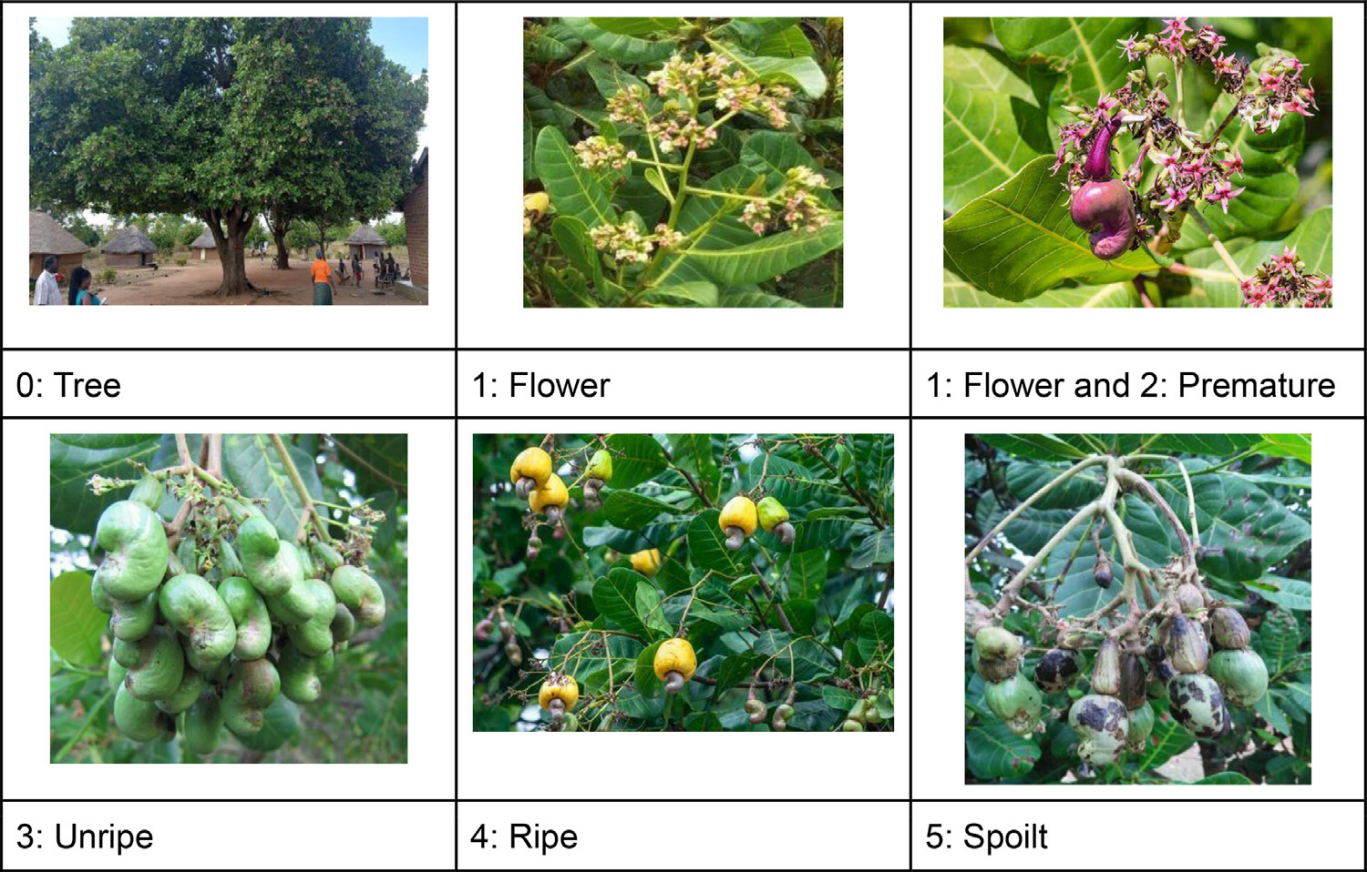
Sample cashew images showing the five class labels.

The majority of the images for coffee capture the full height and breadth of the tree (or plant) from two opposite lateral sides. A few of the images involved imaging the same tree from an overhead position that covers the entire canopy. For cashew trees, images were captured from different lateral sides (no top-view images). The image data for coffee and cashew nuts have been meticulously annotated. These annotated datasets, stored in the YOLO (You Only Look Once) format [6], are now readily accessible on the Hugging Face platform. Tables 1 and 2 provide the number of annotated object instances per class in the coffee and cashew datasets, while Fig. 1, Fig. 2 show sample images for the two crops.

**Table 1:** Number of annotated object instances per class in the coffee dataset.

Object class	# annotated instances
Unripe	121,761
Ripening	630
Ripe	1188
Spoilt	601
Coffee_tree	3060

**Table 2:** Number of annotated object instances per class in the cashew dataset.

Object class	# annotated instances
Tree	5347
Flower	23,169
Premature	21,200
Unripe	5347
Ripe	7481
Spoilt	25,820

The five classes in the coffee image dataset, as shown in Fig. 1, have the following class IDs and labels: 0: Unripe, 1: Ripening, 2: Ripe, 3: Spoilt, and 4: Coffee_tree.

The cashew dataset has six class IDs and labels, as shown in Fig. 2. The classes include 0: Tree, 1: Flower, 2: Premature, 3: Unripe, 4: Ripe and 5: Spoilt.

Our coffee and cashew nut datasets for machine learning yield estimation is the first of its kind and we did not come across any similar publicly available dataset. Existing datasets such as [[7], [8], [9]] consist of coffee leaf images designed for nutritional deficiency and/or plant disease detection and classification. Our dataset was collected using UAV equipment while the studies cited above used smartphone cameras for data collection.

## 3 Experimental Design, Materials and Methods

### 3.1 Field data collection

**Fig. 3:**
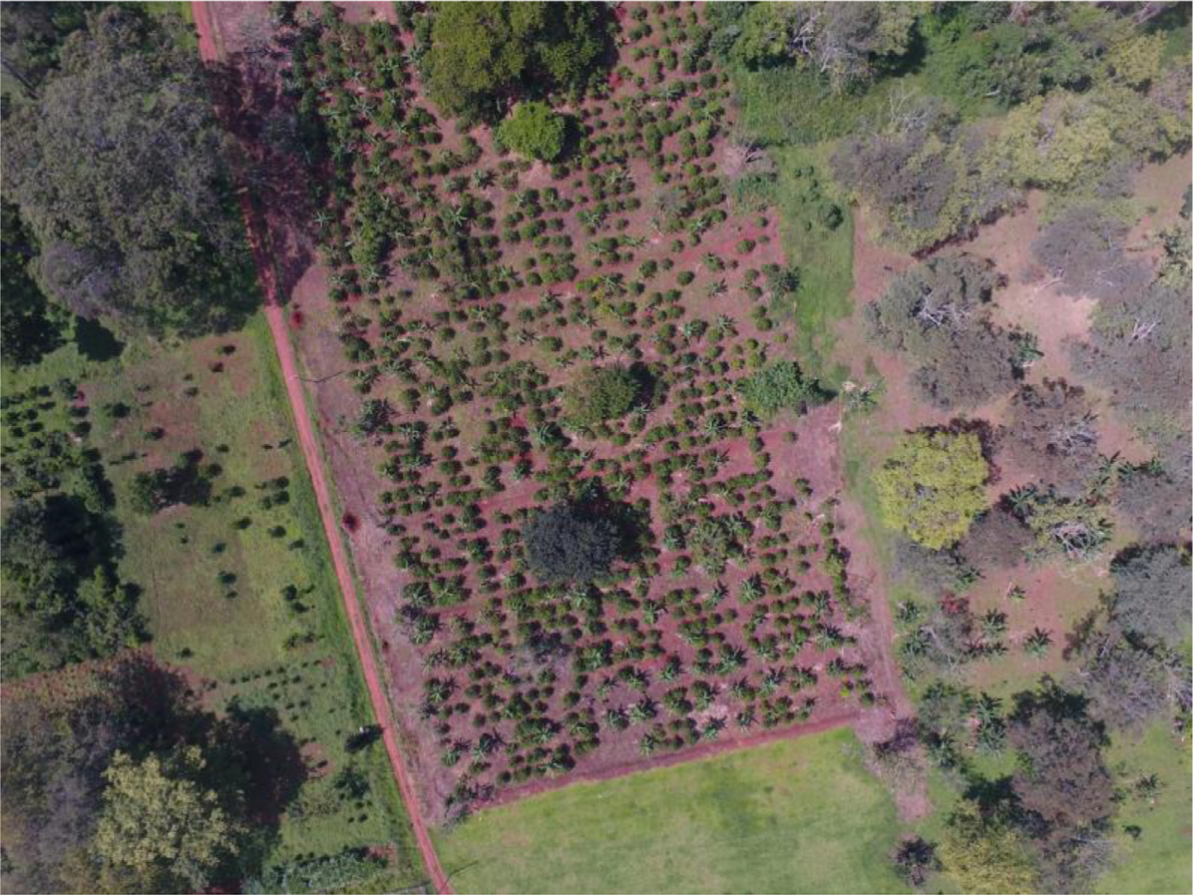
Aerial view of the Block 13 demonstration farm at the National Coffee Research Institute (Latitude 0^o^ 15` 30.696” N and Longitude 32^o^ 47` 25.266” E) where the coffee images were collected.

The datasets were collected from small and medium-scale farms in significant coffee and cashew growing regions of Uganda, including in the southern, central, eastern, and northern parts of the country. However, most of the coffee images were collected from two demonstration farms operated by the Uganda National Coffee Research Institute (NaCORI) in Kituza village, Mukono district, central Uganda. NaCORI is a governmental agency responsible for researching and developing new coffee varieties. Fig. 3 shows one of the demonstration farms (i.e., Block 13) where most of the coffee images were collected. The choice of farms and purposefully sampled plants from which data was collected was advised by agricultural experts who were part of the field data collection team.

The coffee and cashew nut image data was collected in three phases. In the first phase, coffee image data was collected from Bukomansimbi, Kyotera, Mukono, Buikwe, and Masaka districts in Central Uganda between November and December 2022. In the second data collection phase, coffee image data collection took place in June 2023 in the Eastern Uganda districts of Luuka, Jinja, Mbale, and Sironko. In the third data collection phase, cashew nut image data was collected from Lira, Abim, and Nakasongola districts in March 2023. Data collection was carried out during the peak of the harvest season(s) for each crop. The details of images collected per region are shown in Table 3.

**Table 3:** Regions of Uganda where the current datasets were collected.

Region	Crop and Variety	Number of Images
Central, Eastern	Robusta coffee, Arabica coffee	3200
Northern, Central	Cashew nut	3098

## 4 Materials and Methods

Preparatory activities were carried out before field data collection. These included obtaining authorization letters, designing the data collection guidelines, training data collectors, and pilot fieldwork. This was done to prepare the field data collection team, to test equipment and data collection instruments, and to evaluate sample images for quality assurance.

The imaging equipment consisted of a UAV, commonly referred to as a drone. Specifically, we used a DJI Mini 3 Pro drone equipped with a high-quality camera that had a 48 MP 1/1.3 in CMOS sensor, lens with aperture of f/1.7 and focus range of 1 m - ∞, shutter speed of 2-1/8000s and an ISO range of 100–6400 (Auto and Manual).

A custom drone flight strategy was developed and used. This included using manual flight plans, flying at low altitudes and at close distances of about 1 m from coffee and cashew trees, adjusting camera orientation for optimal exposure and visibility of objects of interest, optimal spatial resolution, and flight speed.

**Fig. 4:**
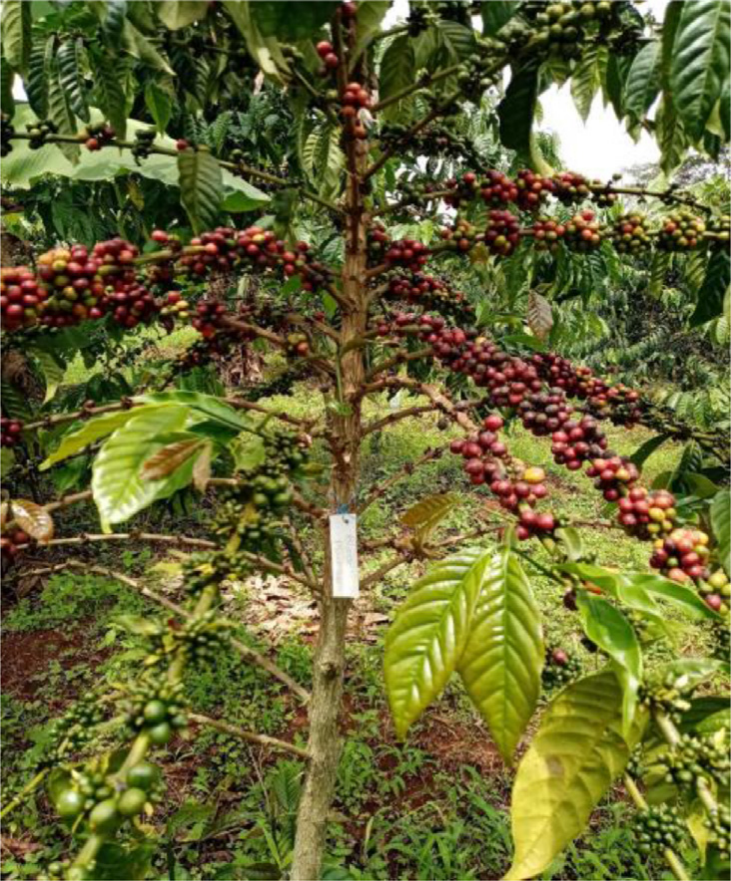
A coffee tree in Block 13 with a label for image data collection.

Images were primarily collected under optimal weather conditions for flying a UAV for farm-based data acquisition, including natural illumination (sunshine), precipitation, temperature, cloud cover, wind speed, and humidity. This was done to ensure that the resulting images were of high quality. Multiple images (at least three) were acquired per each purposively sampled coffee and cashew tree, taken from different viewpoints including from the top and opposite lateral sides. Full tree height and breadth and close range (approx. 1 m) images focused on coffee cherries and cashew apples were acquired (Fig. 4).

### 4.1 Data preprocessing and annotation

**Fig. 5:**
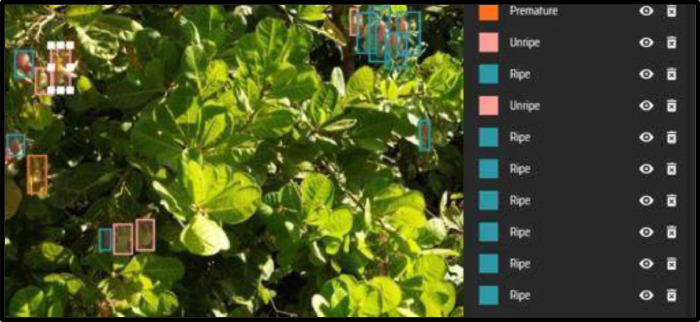
Example of cashew nut image annotation.

We conducted thorough data cleaning, eliminating blurry and overexposed images while resolving any inconsistencies. The cashew data was labelled using an online annotation tool called Makesense AI^1^. The annotated data was saved in YOLO format [7] with six class IDs representing the cashew labels: 0: Tree, 1: Flower, 2: Premature, 3:Unripe, 4: Ripe, and 5: Spoilt based on a categorization in [10]. Fig. 5 shows an example of cashew nut image annotation.

**Fig. 6:**
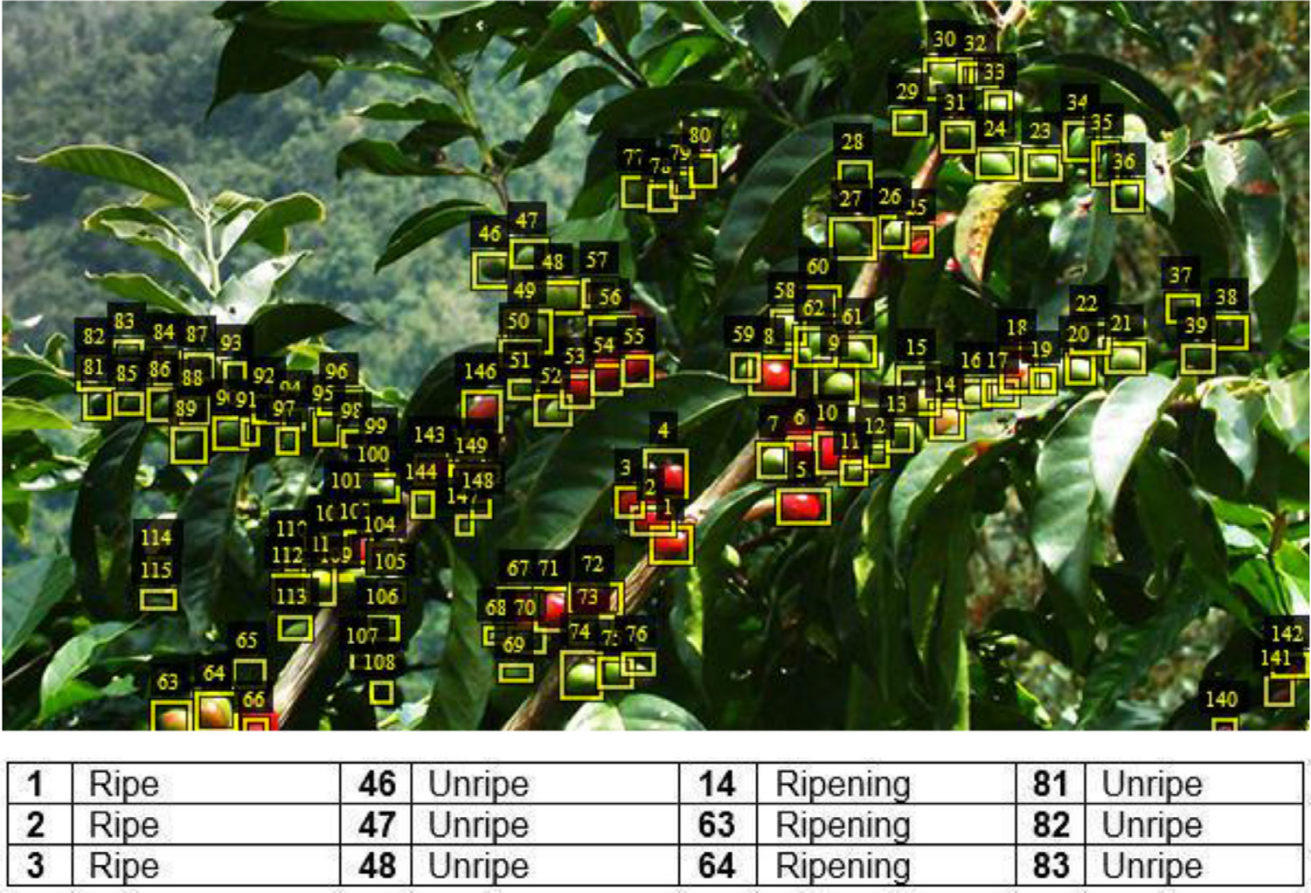
Example of coffee image annotation.

For the coffee image data, the coffee specialist from NaCORI expertly handled the annotation process using an offline tool called VGG Image Annotator (VIA) [[8], [11]] to annotate the images. The coffee annotated data was saved in YOLO format with 5 class IDs representing the coffee labels: 0:Unripe, 1:Ripening, 2:Ripe, 3:Spoilt, and 4:Coffee_tree based on categorisation in [12]. Fig. 6 shows an example of coffee image annotation.

## CRediT authorship contribution statement

**Rahman Sanya:** Conceptualization, Data curation, Project administration, Writing – original draft. **Ann Lisa Nabiryo:** Data curation, Validation, Writing – original draft. **Jeremy Francis Tusubira:** Data curation, Validation, Writing – original draft. **Sudi Murindanyi:** Data curation, Writing – original draft. **Andrew Katumba:** Funding acquisition, Supervision. **Joyce Nakatumba-Nabende:** Conceptualization, Supervision, Funding acquisition, Writing – review & editing.

## Data Availability

Coffee and Cashew Nut Dataset (Original data) (Mendeley Data)

## Footnotes

1 https://www.makesense.ai/.
